# Facilitating personal development for public involvement in health‐care education and research: A co‐produced pilot study in one UK higher education institute

**DOI:** 10.1111/hex.13097

**Published:** 2020-07-24

**Authors:** Sue Read, Alison M. Aries, Sue M. Ashby, Val Bambrick, Steven J. Blackburn, Helen Clifford, Carol Rhodes, Sarah Thirlwall, Carole A. Watkins

**Affiliations:** ^1^ Learning Disability Nursing Keele University Staffordshire UK; ^2^ Physiotherapy and Rehabilitation Science Keele University Staffordshire UK; ^3^ Nursing Keele University Staffordshire UK; ^4^ Keele University Staffordshire UK; ^5^ Research Institute for Primary Care and Health Sciences Keele University Staffordshire UK; ^6^ School of Medicine Keele University Staffordshire UK; ^7^ Research Institute for Primary Care and Health Sciences Keele University Staffordshire UK

**Keywords:** action research, co‐production, engagement, personal development, qualitative research

## Abstract

**Background:**

Public involvement in the education of students enrolled on higher education programmes has gained impetus. For students enrolled on professional health‐care programmes and health‐related modules in the UK, there is also a requirement by professional bodies to include “service user” involvement in preparation for entry to a professional health‐care register and continuing professional development. Actively involving patients and members of the public in research is also a requirement by many research funders. In this article, the term Patient and Public Involvement (PPI) will be used throughout to include lay members, volunteers, user and carers.

**Objectives:**

A unique pilot study was introduced across a health faculty to integrate PPI in a deliberate way. It aimed to provide an educational, focused programme of events that was meaningful to develop and inform peoples’ knowledge, skills and confidence for their involvement in the health faculty.

**Design:**

PPI members volunteered to sit on a steering group to determine the educational journey; the outcomes of three focus groups with PPI members (N = 32) and academics informed the programme content which included a range of workshops covering the exploration of public roles and barriers to involvement, introduction to research and interviewing skills.

**Results:**

The workshops were well attended, and outcomes indicated the importance of co‐production when designing, delivering and evaluating programmes.

**Discussion:**

Co‐production underpinned this pilot study, resulting in a programme which was meaningfully received by public contributors.

**Recommendations:**

Co‐production was seen as integral to this research to ensure that outcomes were indeed “fit for purpose”.

## 1 INTRODUCTION, BACKGROUND AND RATIONALE

“…people experience illness within a narrative, or story that shapes and gives meaning to what they are feeling, moment to moment. Illness narratives are noticeable because they are usually different from those of the rest of our lives”^1^ (Donald, 1998: 17)

In the United Kingdom (UK) and other developed countries, public involvement in the teaching and learning of students enrolled on higher education programmes has gained impetus. This has been driven by the requirement of higher education institutions (HEIs) to develop a future workforce that is able to engage with (and be responsive to) the environments (and those who work within them) in which they intend to seek future employment.^2^ HEIs embrace a variety of activities which foster the qualities of leadership, responsibility, personal integrity, empathy, care and respect for others, accountability and self‐regulation which are reflected in respective university graduate attributes.^3^

For the purpose of this paper, the terms Patient and Public Involvement (PPI) and public contributors will be used throughout to include lay members, volunteers and user and carers. The role of PPI in raising awareness of issues that are important to them, in making connections with real‐life situations and the impact of processes, systems and interactions with others, is acknowledged as significant and powerful when preparing students for roles they aspire to successfully achieve in society.^4^

There has been a significant shift to involving patients and the public in the process of health‐care research with organizations such as INVOLVE providing comprehensive guidance.^5^ Major research funders now require credible evidence to demonstrate how patients and the public have contributed to any research proposal and to the delivery of studies.^6^ Indeed, from a research perspective, the Public Involvement Standards Development Partnership^7^ have recently introduced six key standards “…for people and organizations that do research, support research and do public involvement to improve research”..^7^ This UK partnership incorporated national health research funding organizations (Chief Scientist Office (Scotland), Health and Care Research Wales, the Public Health Agency (Northern Ireland) and the National Institute for Health and Research (England), who collectively developed and launched the Standards. These six standards are identified in Table 1. They encapsulate much of the thinking that many HEIs have established by developing research “user” groups working with researchers, academics and clinicians; responding to the challenges of achieving and sustaining active and meaningful public involvement in research.^8^

**Table 1:** National Standards for Public Involvement in Research^7^

Standard 1: Inclusive opportunities	We offer public involvement opportunities that are accessible and that reach people and groups according to research needs.
Standard 2: Working together	We work together in a way that values all contributions, and that builds and sustains mutually respectful and productive relationships.
Standard 3: Support and learning	We offer and promote support and learning that builds confidence and skills for public involvement in research.
Standard 4: Communications	We use plain language for timely, two way and targeted communications, as part of involvement plans and activities.
Standard 5: Impact	To drive improvement, we capture and share the difference that public involvement makes to research.
Standard 6: Governance	We involve the public in our governance and leadership so that our decisions promote and protect the public interest.

For students enrolled on professional health‐care programmes and health‐related modules in the UK, there is also a requirement by professional bodies to include “service user” involvement in preparation for entry to a professional health‐care register and continuing professional development, for example doctors, nurses, allied health professionals.^9^, ^10^, ^11^ The term “service user” is reflected within these regulatory frameworks and for the purpose of this paper is defined as individuals and their surrounding support, for example partners, family, informal carers. This is an acknowledgement of service user expertise as “experts by experience”,^12^, ^13^ to facilitate students in achieving care which sustains a focus on meeting the needs of the service user within the challenging environment of contemporary health care.^14^, ^15^

Public contributors are involved in the recruitment of health‐care students to pre‐entry programmes, consultation (entry criteria, content, process and validation of programmes), delivery and assessment of teaching and learning, the evaluation of content and other monitoring of student processes, for example Health and Conduct Committees. There has been a plethora of literature relating to how public contributors are involved in these activities and the benefits of doing so.^16^, ^17^, ^18^ There has also been a considerable increase of available guidance for professionals wanting to involve service users in health‐care education (eg ‘Working Together^19^, ^20^ The Service User and Carer Toolkit—http://www.serviceuserandcarertoolkit.co.uk/principals.html).

HEIs providing health‐care education are influenced by the drivers of change within health‐care provision, one of which is “co‐production” described in 2012 as “*the new role of citizens and civil society*”.^21^ Co‐production in health care is defined as “*a way of working that involves people who use health and care services, carers and communities in equal partnership; and which engages groups of people at the earliest stages of service design, development and evaluation”* ^22^ and involves a long‐term relationship. Co‐production was highlighted in the “Realising the Value” campaign which identified the importance of enabling people to take responsibility for their own health and to be active in working with communities and their resources,^23^ whereas co‐production from a research perspective “… *is an approach in which researchers, practitioners and the public work together, sharing power and responsibility from the start to the end of the project, including the generation of knowledge. The assumption is that those affected by research are best placed to design and deliver it and have skills and knowledge of equal importance”*.^7^ The role of co‐production in research is gaining interest with the publication of recent NIHR INVOLVE guidance.^7^ Co‐production was seen as integral to the research presented in this paper, when designing and developing educational programmes in conjunction with public contributors, for public contributors and co‐delivered by academics and public contributors in order to ensure that outcomes were indeed “fit for purpose”.

Within this HEI, members of PPI had received training, knowledge and some skills from the individual School's already but what was unique to this programme was delivering training across the Faculty and bringing people together across the range of differing roles. This was specifically important on three counts:

- Recognizing that PPI members felt quite isolated and did not seem to know what other roles were available and what else was going on in the university.
- Acknowledging that there are common skills needed across the varied roles.
- Identifying potential efficiencies in delivering at faculty level rather than School levels only.

Whilst PPI is now an essential aspect of health‐care education and research, there is minimal guidance available for public contributors to prepare them for the variety of roles and activities this involves.^24^ There are few formal training courses outside of individual institutions or organizations. INVOLVE are developing a repository of training courses for research involvement available nationwide (http://www.invo.org.uk/resource‐centre/learning‐and‐development/whats‐on‐this‐website/), but this will not as yet include a central assessment of content or quality.

This pilot study addresses the identified gap in knowledge of PPI members within an established University in the West Midlands, affirming the need for information and support to inform their existing (and developing) roles. Personal development means different things to different people; INVOLVE advocate that staff in organizations has access to staff development to help develop their knowledge and skills yet members of the public do not always have access to organizational or informal learning opportunities and might require a more concerted approach to meet their developmental needs.^25^

It recognized the need to appropriately prepare service users to meaningfully and effectively maximize their expertise in informing the education of health‐care professionals. Embracing co‐production^22^ and research guidance, public contributors were involved in the full research cycle^5^; from consultation, development of topic guides, data collection, data analysis, through to dissemination, implementation and evaluation of impact. According to INVOLVE,^26^ Involvement in research refers to active involvement between people who use services, carers and researchers, rather than the use of people as participants in research (or as research “subjects”). Alternatively, Participation includes taking part in a research study, for example people being recruited to take part in a clinical trial or another kind of research study, joining in a focus group or completing a questionnaire, whereas Engagement is recognized as where information and knowledge about research is provided and disseminated, for example science festivals, open days, media coverage. PPI members were involved throughout this research form initially establishing the programme, to co‐delivering the programme and dissemination on completion.

There are also different types of co‐production. The NCAG has a ladder of co‐production but this is used to describe co‐production at a strategic commissioning level. In research, we would describe it as being involved from defining the research question right to implementing the research into practice. The overwhelming tenant about co‐production is that it involves a long‐term relationship.

### 1.1 Research aims

The aims of this research were to identify, develop, introduce, deliver and evaluate a programme of personal development for PPI involved educational and research activities across a Faculty of Medicine and Health Sciences, which incorporated Research Institutes and Schools of medicine, nursing and rehabilitation.

Whilst individual School's already incorporated training for specific groups of public contributors, the intention was to further support those who already dedicate their time to enhance the work of a Health Faculty, and to encourage others to think about doing so. This programme would ensure that public contributors could learn about each other and the work that goes on at the host University. It would provide opportunities for people to share their skills; learn new skills; and to learn about the Faculty specifically and the wider University more generally.

### 1.2 Ethical Considerations

No research involving human participation is without ethical considerations. The host institution provided independent ethical scrutiny and subsequent approval for this research study (Ref No. ERP320). Informed consent was obtained from participants prior to any data collection and all data were stored in accordance with the 1998 Data Protection Act (Great Britain, 1998).^27^ Anonymity of participants was maintained during dissemination of the information from the study.

### 1.3 Methods and procedures

**Figure 1:**
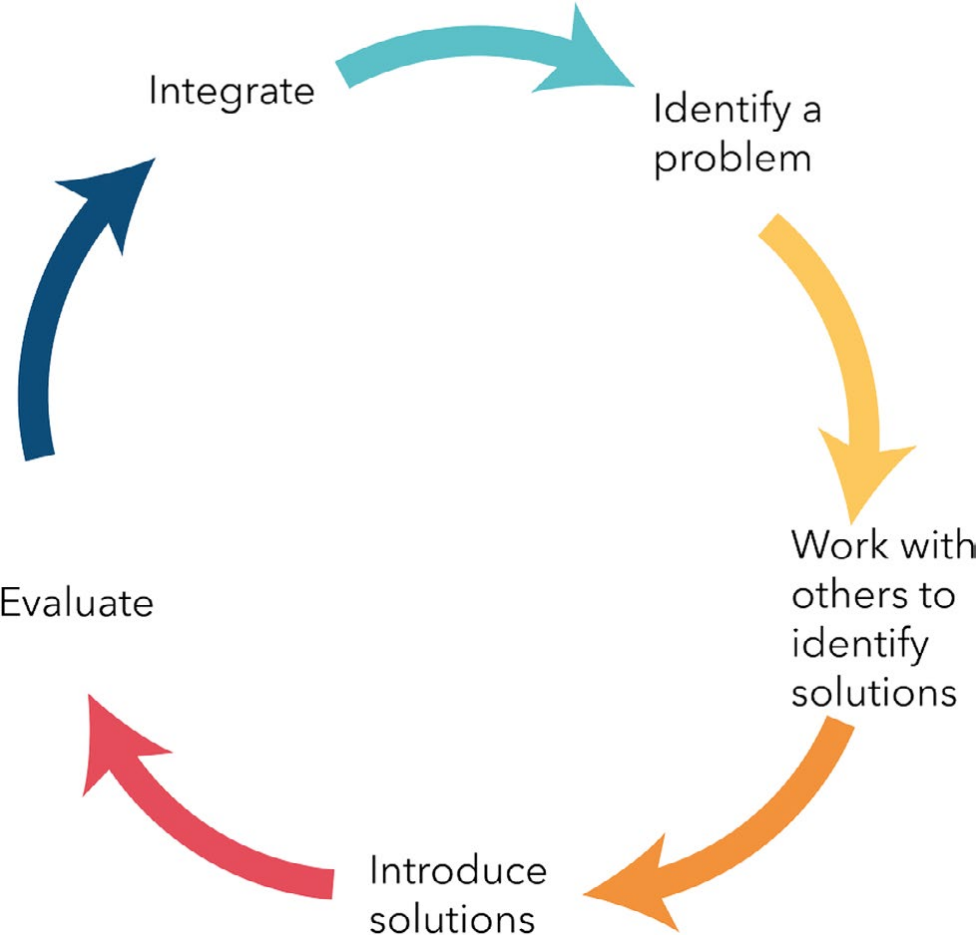
The action research cycle

An action research approach was adopted for this research study. Participatory Action Research projects usually have the underlying tenets of a collective commitment to investigate an issue or problem; a desire to engage in self‐ and collective reflection to gain clarity about the issue under investigation; a joint decision to engage in individual and/or collective action that leads to a useful solution that benefits the people involved; and the building of alliances between researchers and participants in the planning, implementation and dissemination of the research process.^28^ PAR is fundamentally a cyclical process, alternating continuously between enquiry and action, and between practice and innovative thinking.^29^ This alternating process actively enables the implementation of change and the subsequent generation of theory.^30^ These aspects are all imperative to the action research process as illustrated (see Figure 1).

“*Increasing importance is being placed on service users and professionals being partners in services*….”^31^ and creative ways of such engagement remains important to this involvement. It has been suggested that action research as a means of cooperative investigation enables research to be undertaken in collaboration with the people involved.^32^ In this particular research study, the empowering and emancipation element of action research^33^ necessitated a collective, self‐reflective inquiry and provided the opportunity and time to consult and discuss with a number of university‐based academics and public contributors across a Health Faculty from an educational strategic and operational perspective.

This particular research study incorporated several phases across the general participatory action research (PAR) framework.^34^ Members of the PPI groups were actively involved in formulating and identifying educational topics, programme development, programme delivery, action design, implementation, evaluation and research dissemination.^35^ Such co‐productivity was perceived as fundamentally instrumental throughout this study^36^ in keeping with PAR processes.

### 1.4 Inclusion criteria for study

At the time of this study, the Faculty of Medicine and Health Sciences, of the host institution was comprised of four Schools (Schools of Health and Rehabilitation (SHAR); Nursing & Midwifery (SN&M); Pharmacy; and Medicine (SoM) and two Research Institutes (RI of Primary Care and Health Sciences; RI Science, Technology and Medicine).

Inclusion to this study was all adult PPI members across the Faculty; other PPI members outside of the Faculty were excluded from participation and the invitation to participate reflected this.

### 1.5 Phase one—Identifying the problem

The Faculty of Medicine and Health Sciences’ User and Carer Liaison Group had been established for five years at the onset of this study. The group consisted of academics with a responsibility or interest in PPI, from across the faculty and one public contributor member. The aims of this Faculty group were to:

“…consolidate and strategically drive the user and carer activities and developments across the Faculty. It will provide advice and assist Schools and RIs to further develop user and carer involvement in University business through providing regular forums for communication, discussion and healthy debate and to disseminate all outcomes across the Faculty. It will also link in with other university groups to promote a formal integration of ideas and communication” (internal document, 2018)

As part of this group's work, a number of Faculty wide issues were critically explored, and one aspect identified by academic members was that of concerns relating to the educational and developmental support of public contributors across the Faculty. A Steering Group of members of the Faculty group, including three public contributors, was subsequently formed to develop, analyse and evaluate training opportunities, which took this work forward in a deliberate fashion.

### 1.6 Phase two—Working with others to identify solutions

Co‐productive working was key to these processes throughout, starting with focus groups to ascertain education and training needs. Three focus groups (n = 9; n = 14; n = 9) were undertaken facilitating an exploration of opinions and experiences that could be shared and detailed.^37^ The aim of these focus groups was to obtain qualitative data from public contributors who had experience working across the Faculty of Medicine and Health Sciences of the host HEI pertaining to education and training opportunities from a content and practical perspective.

Purposive sampling was used to ensure that all participants met the inclusion criteria and had experience of working ^within^ the relevant Schools or RI’s at the host HEI. This ensured that the participants had the relevant knowledge to participate in the focus group discussions.^38^ Participants were recruited from the databases detailing the public contributors currently involved across the Faculty. PPI members affiliated to the specified Schools and Faculties were contacted via telephone, email or post to ask if they were interested in participating in this study. Those individuals who expressed an interest were sent an information sheet about the study, informing them about the focus groups. Participants were given at least 48 hours to decide if they wish to partake, prior to consenting and participating in the focus group.

The focus group methodology enabled group discussions to be undertaken, generating large amounts of rich data. This allowed for small groups of people who were able to discuss ideas with the other group members.^39^ Three researchers (member of the team/ authors) facilitated the focus group discussions. Three focus groups were held (n = 32); key questions and probes were used, as recommended by Krueger and Casey,^37^ following the development of a topic guide a priori, to ensure discussion in the focus groups related to the topic area of the training needs of the PPI groups. Discussions were recorded via a digital recorder.

The recordings were transcribed verbatim and independently analysed by three members of the research team using thematic analysis, a recognized categorizing strategy for qualitative data. By then discussing the outcomes, consensus agreement enabled researchers to move their analysis from a broad reading of the data towards discovering patterns and developing themes.^39^ The findings of phase two directly informed the educational programme for the public contributors within the Faculty of Health Schools and RI’s.

### 1.7 Phase three—Introduce the solutions

After completion of phase two of the study, public contributors who had attended the focus groups were invited to a consultation workshop (n = 27) where further discussion enabled a consensus agreement of the main issues to be included in the proposed training programme. Participants were also invited to join academics as part of the Steering Group to collaborate with the researchers over several meetings to further develop and refine the proposed programme. This resulted in a small group of public contributors (n = 5) working alongside academic partners to deliver the nine topics using a range of different delivery styles.

PPI members across the Faculty were then invited to attend the training programme which ran from May 2016 to February 2017. All participants could select pertinent sessions.

### 1.8 Phase four—Evaluation

Attendees were requested to complete a post‐sessional evaluation questionnaire, which was deliberately quick and simple to complete and individualized to the content of the training session. The questionnaire incorporated 16 areas of free text and Likert scale questions. A data capture sheet was developed and introduced for each session, providing a consistent approach to analysis of the evaluation data (see Table 3) The responses to these evaluation questionnaires were analysed utilizing both quantitative (eg overall score for each session) and qualitative methods (thematic analysis). The responses from the PPI members informed the overall evaluation of the training programme.

### 1.9 Phase five—Dissemination and integration of the information

On completion of the training programme, a celebratory event was held to thank everyone for their contributions and to share the findings of the evaluation. All those involved in the workshops, focus groups and training programme were invited. PPI members and academics jointly designed, organized and facilitated this event, continuing with our co‐production focus. This included jointly identifying the content and format of the day's events, incorporating three presentations from the perspective of public contributors. All feedback and suggested amendments to the existing programme were considered prior to any further implementation of any future programmes.

**Figure 2:**
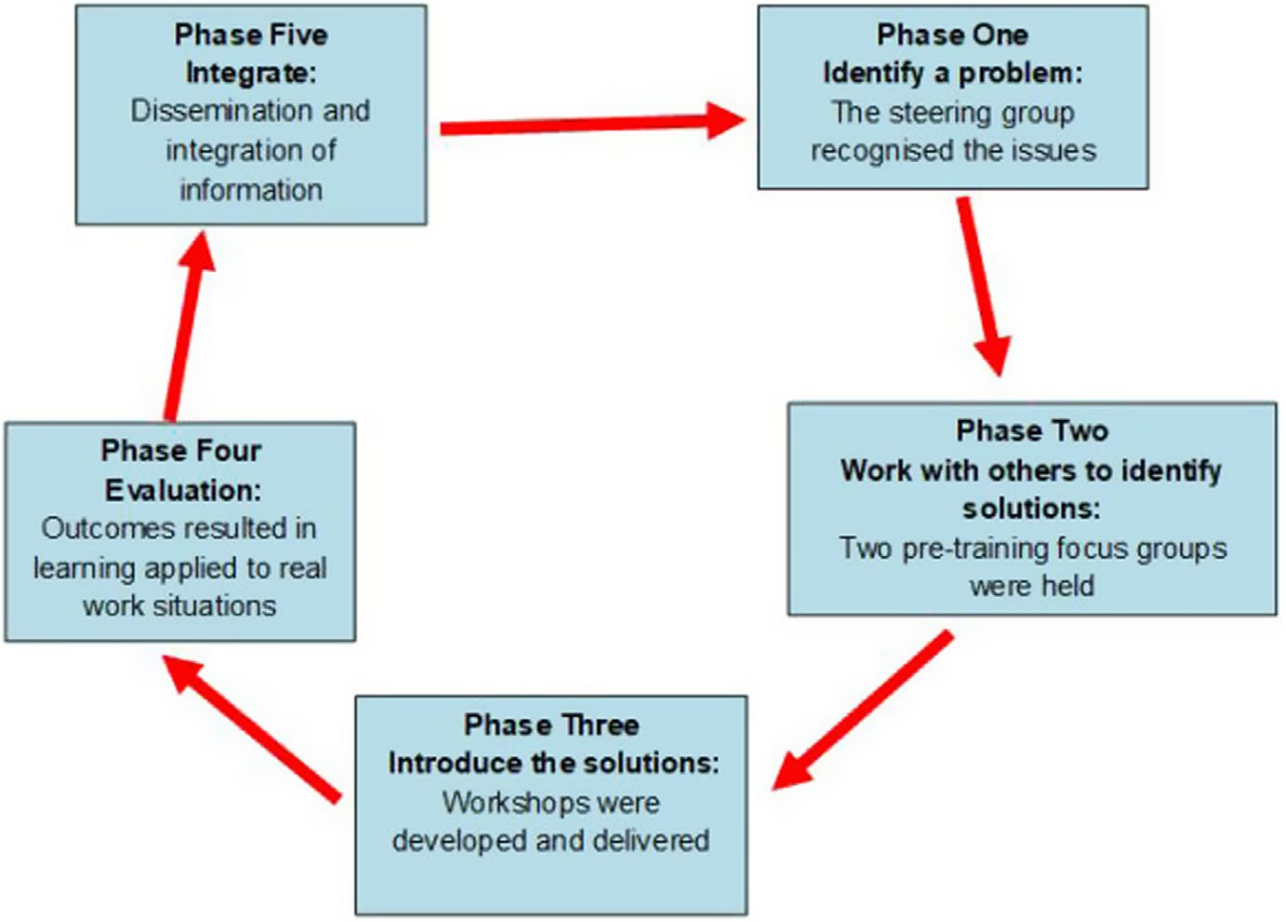
Research processes in the PAR framework

An overview of the research process as it evolved in conjunction with the PAR process is found in Figure 2.

## 2 RESULTS

### 2.1 Identifying the problem, introducing the solution: The learning and development programme

Nine half day sessions were held which were all co‐facilitated by both academic and public contributors. This co‐facilitation throughout the programme was a fundamental principle agreed at the start of the process. The sessions included a general introductory session; exploration of roles and barriers; importance of personal stories; introduction to research; communication sessions (written, verbal/non‐verbal/information technology); participation in meetings; equality and diversity; health and safety; and interviewing skills. A variety of approaches to deliver the sessions were used, with a predominant interactive focus responding to the needs of the individuals attending the sessions. For example, during the patient stories session, the active sharing of one's own story was encouraged to reinforce the value of storytelling, what can be learnt and how to support both the story teller and story gatherer. The session on research explored how members of the public can be involved throughout a research project, from its inception to its dissemination. Participants learnt about the research process and, with real‐life examples to prompt discussion, considered the role and value of public involvement in different stages of a research project.

### 2.2 Programme evaluation

#### 2.2.1 Participant evaluation

The number of participants attending the training sessions was variable (see Table 2). A total of 69 participants attended across the 9 sessions, with a core group of three participants attending all of the workshops. There were significantly more females attending than males, but this directly reflects the ratio of those who dedicated their time to the University. With such a wide diverse group involved in so many different types of involvement across the university, a number of key sessions were consistently attended, whilst some were specifically selected by the participants.

**Table 2:** Summary of attendance

Title of session	No of attendees		
	F	M	Total
Introduction to host institution	5	1	6
Exploring roles and barriers to taking part	8	2	10
Recognising the value of personal stories/experiences	8	2	10
The importance of good communication	4	2	6
Introduction to research	6	1	7
Participating in meetings	5	2	7
Equality and Diversity Training/Health and Safety	7	4	11
IT for beginners	2	2	4
Interviewing skills	5	3	8
Total number of attendees	50	19	69

#### 2.2.2 Participant feedback

Table 3 presents a summary of the participants responses to the evaluation questionnaire completed after each session of the programme. All evaluations were anonymous, hence we cannot ascribe names or frequency of the quotations used to substantiate text.

Having contributions valued remains important, and whilst the majority of the sessions were well evaluated, participants particularly welcomed having their views shared and heard (agree 11; strongly agree 41).

**Table 3:** Overall evaluations

Question	Strongly disagree	Disagree	Agree	Strongly agree
Session encouraged me to share my views of being involved in the host institution	0	1	11	41
Session encouraged me to influence the development of public contributors’ engagement at the host institution	0	1	18	34
Topics explored were appropriate and relevant	0	0	13	40
I felt able to contribute to discussions	1	1	10	42
I feel adequately prepared for contributing on the day	0	1	19	33
I found the content interesting and useful	0	0	12	38
I was happy with the information received before the day	0	2	20	25
I was happy with the refreshments on the day	0	0	14	32
The session ran smoothly	0	0	13	36
I enjoyed the session	0	0	11	39
I felt my contribution was valued	0	0	13	36
I feel that I benefitted from the session	0	0	10	40
I would recommend the session to others	0	0	11	39

“It has been informative and I feel valued as a lay rep” (Participant)

The highest scored point was about feeling able to contribute to discussions and sharing experiences (agree 10; strongly agree 42).

“Welcomed opportunity to hear the experiences of other lay people” (Participant)

“It also gave me an insight into the diverse range of backgrounds of the lay advisors” (Participant)

Most participants felt well prepared for the day (agree 19; strongly agree 33), with just two participants disagreeing. Everyone felt that their contributions were valued, that they did indeed benefit from the session, and would recommend the session to others:

*“*My role is evolving and being at ….will enhance my understanding and roles” (Participant)

“Confident that my contribution was valued” (Participant)

The results illustrated that the programme was welcomed by participants, and interestingly a number of the participants who had contributed to the work at the University for many years, and yet still knew very little about the wider University, the roles of people involved and what it had to offer them, as participants said:

“Roles at …have been more defined and I now know what to expect when attending meetings” (Participant)

“I have been able to update my skills and I will be able to pass on my knowledge to others” (Participant)

One interesting suggestion included involving volunteers more in the delivery of the programme, thus relieving academic staff of this intense labour investment. Participants appeared to recognize the importance of the programme, and how it would enhance their current role:

“Future development and involvement will support my role” (Participant)

“Totally informed and lots of knowledge” (Participant)

“Amazing amount I have learned today” (Participant)

### 2.3 Dissemination event and integration of suggestions

All the people who were involved with the workshops, focus groups and training programme were invited to the end of study celebration event. There was a reasonable attendance of 31 participants. There was a general consensus that the programme was a success and many people were supportive of continuing with it. Following the presentations of the study findings and learning gained, the participants discussed how the programme could be improved and what might be needed to sustain it.

## 3 DISCUSSION

This was the first time that such an educational programme had been delivered across the Faculty at the host HEI for PPI members, so it was unique in this respect, and is likely to be unique in other similar organizations. Overall, the programme was well attended and extremely well evaluated by both participants and facilitators (see Tables 2 and 3). Educational preparation for any new programme is labour intensive, and in this case it was particularly difficult since it was a cross Faculty initiative, and those academics involved had no dispensation for the work it entailed, having to fit it in alongside their existing work commitments. Preparation for the PAR study was particularly lengthy, including: difficulties in co‐ordinating meetings with academics and PPI members; helping colleagues to appreciate the inherent PAR process.

The preparation of the programme incorporating the consultation with public contributors was extensive and labour intensive (12 months). But, genuine co‐productive working takes time to build relationships, manage meetings of busy people; using supportive communication, and involving the right people.^40^ We talked with, and listened to, a number of public contributors across the Faculty who helped to shape and deliver the programme to meet their perceived needs.

Co‐production was integral to the development, delivery and dissemination of this programme. As a delivery model for health services, co‐production is based on the sharing of information and on shared decision making between the service users and providers.^41^ It builds on the assumption that both parties have a central role to play in the process as they each contribute different and essential knowledge.^42^, ^43^ PPI members were involved throughout this work, and the results well illustrate their satisfaction regarding their involvement.

However, it was a very cost‐effective programme overall, since costs were largely absorbed within Schools as academic facilitators were all from the Faculty. Additional costs mainly related to travel and refreshments, and were met by a small Faculty budget. We evidenced many examples of the value of the programme:

“I was fairly ignorant of interview process and lay involvement” (Participant)

Participants recognized that it was about going further than just the programme as they commented that:

“Reinforcement of the value of community involvement” (Participant)

“Hope the course can continue” (Participant)

“An excellent use of my time” (Participant)

The programme strengthened the value and importance that the University placed on the involvement of public contributors, and this too was recognized:

“Please make this an ongoing approach” (Participant)

The importance of PPI in research has never been stronger, yet there are few studies that measure explicit impact.^44^ Due to staff and PPI members movement, follow up explorations regarding longer term impact were not conducted, which can be seen as a disappointing limitation to this study.

Should the programme continue, careful practical considerations regarding the timings (when, where and how sessions should be presented) and content of the programme requires careful thought and many discussions in keeping with the co‐productive approach. The PAR approach to programme development is worthy of replicating in this particular context.

## 4 CONCLUSIONS

Including public contributors in all aspects of health‐care programmes at a busy University in the West Midlands was an accepted important ingredient to its educational and research programmes. However, identifying creative ways to ensure continuing commitment in a meaningful way proved challenging. A pilot study was introduced across a Health Faculty to integrate PPI in a deliberate way and to provide an educational programme of events that were meaningful and appropriate. The aims of this research were to identify, develop, introduce, deliver and evaluate a programme of personal development for PPI involved educational and research activities across a Faculty of Medicine and Health Sciences, which incorporates Research Institutes and Schools of medicine, nursing and rehabilitation. Qualitative approaches to data collection and analysis worked well when meaningfully engaging with a range of people outside of the general university environment.

Co‐production was a deliberately selected approach that underpinned this pilot study, resulting in a programme which was meaningfully received by service users. It illustrated well a useful framework for development programmes explicitly for public contributors, deliberately benefitting all stakeholders. Dovetailed against focus groups, both approaches worked really well.

Universities should invest time, effort and resources in supporting public contributors in order to get the ultimate engagement from a range of volunteers and to affirm their importance to educational programmes and when conducting research.

“Totally informed and lots of knowledge” (Participant)

The proposal by some participants for greater involvement of public contributors in delivering future programmes suggests a recognition of the time and effort spent by academic staff in running the programme. Indeed some participants specifically articulated their awareness of the degree of commitment from staff, both in terms of time and preparation/delivery. In addition, the co‐production process seems to have provided those participants with the confidence to feel able to take on additional responsibilities in future iterations of the programme. Realpe and Wallace^45^ argue that for it to “be truly transformative, co‐production requires a relocation of power towards service users” and the dissemination event demonstrated that some participants felt more empowered to take forward/lead on elements of future programme delivery. Such approaches to deliberate social inclusion of PPI members are more likely to be appropriate for involving seldom heard or difficult to reach populations such as those with an intellectual disability or mental health diagnosis.^46^

Whilst the value of service user expertise as “experts by experience”^12^, ^13^ is central to health‐care education and research it can be a challenge to recruit users and carers or to ensure diversity of recruitment. In addition those involved may be affiliated to only one School/Department within a Faculty, although all learning and development programmes are inclusive and accessible to support a diverse PPI community. As such, public contributors may have a limited perspective of the potential roles available for them within health‐care education, and HEIs may not be able to fully utilize the range of knowledge and experience their users and carers can offer. The programme provided participants with the opportunity to share their own particular knowledge and experience with others across the Faculty. It offered a potential mechanism to encourage participants to explore other opportunities for involvement beyond their current roles, as well as further validating participants’ own current involvement. Additionally, the increased awareness of other available roles may serve as a means of facilitating further recruitment as those involved in the programme share their gained knowledge with others. However, discussions prompted involving the wider university that is social scientists and the wider implications for citizen engagement and the continuum of business of the University with a focus on health and well‐being and establishing relationships with local communities.

“Well done team” (Participant)

## Data Availability

The data that support the findings of this study are available from the corresponding author upon reasonable request.
